# Determinants of Length of Hospital Stay in Older Adult Hip Fracture Patients in a Northern Peruvian Hospital

**DOI:** 10.3390/jcm14238564

**Published:** 2025-12-03

**Authors:** Edwin Aguirre-Milachay, Bryam William Sarmiento Llaguenta, Jesús Manuel Verona Mendoza, Darwin A. León-Figueroa, Mario J. Valladares-Garrido

**Affiliations:** 1 Hospital Nacional Almanzor Aguinaga Asenjo, EsSalud, Chiclayo 14001, Peru; edwinh.aguirre@gmail.com; 2 Facultad de Medicina Humana, Universidad de San Martín de Porres, Chiclayo 14012, Peru; bryam_sarmiento@usmp.pe (B.W.S.L.); jesus_verona@usmp.pe (J.M.V.M.); darwin_leon@usmp.pe (D.A.L.-F.); 3 Hospital Nacional Sergio E. Bernales, Lima 15324, Peru; 4 Escuela de Medicina Humana, Universidad Señor de Sipán, Chiclayo 14001, Peru

**Keywords:** hip fractures, frail elderly, length of stay

## Abstract

**Background/Objectives:** Hip fracture is a condition with increasing hospital demand, and the determinants of hospital stay are crucial for improving clinical outcomes and costs in this vulnerable population. To establish the determinants of the length of hospital stay (HS) of older adult patients with hip fractures in a hospital in the Lambayeque region of Peru during 2017–2019. **Methods:** We conducted an observational study based on a secondary data analysis. The outcome variable was HS, measured in terms of days from admission to the hospitalization unit until discharge. The main independent variables were age, functional ambulation category scale, cognitive status index (Mental Red Cross scale), Barthel index, comorbidities, geriatric syndromes, trauma diagnosis, reason for surgical delay, preoperative time and preoperative complications. We performed a Poisson or negative binomial regression through crude and adjusted models. **Results:** Of 399 patients, the average age was 82.25 years, with 63.7% being female. A Poisson and negative binomial regression analysis were conducted for the variables that were significant in the crude model, which were sex, multimorbidity, mental Red Cross scale, Barthel index, functional ambulation, number of geriatric syndromes, traumatic diagnosis, reason for delay in the first model, preoperative complications in the first model, emergency stay, and preoperative time in the second model. According to the adjusted model, the analysis found that in the first model, advanced dementia as measured by the Mental Red Cross (MRC) scale was associated with an increase in hospital length of stay (IRR = 1.82, 95% CI = 1.03–3.23, *p* < 0.04); similarly, having preoperative complications increased hospital length of stay (IRR = 1.56, 95% CI = 1.30–1.86, *p* < 0.001), adjusted for clinical variables; in the second model, preoperative time was associated with an increase in hospital length of stay (IRR = 7.44, 95% CI = 6.96–7.96, *p* < 0.001), adjusted for emergency department stay. A third global model was developed, finding that advanced dementia as measured by the MRC (IRR = 1.82, 95% CI = 1.02–3.23, *p* < 0.04) and the presence of preoperative complications (IRR = 1.56, 95% CI = 1.30–1.86, *p* < 0.04) were associated with increased hospital length of stay, adjusted for clinical and hospital variables. **Conclusions:** The average HS of older adult hip fracture patients treated at a tertiary hospital in the Lambayeque region of Peru was 17 days. The main determinants of HS duration were advanced dementia and presence of preoperative complications.

## 1. Introduction

A hip fracture is when one of the bones that form the hip joint breaks [1]. This condition is characterized by the disruption of bone continuity, which can cause pain, limited mobility, and functional impairment in the affected person [2,3]. In 2021, the global incidence of hip fractures reached 16.9 million, with an increase of over 126% since 1990, with rates of 182 per 100,000 inhabitants and greater frequency in older adults [4]. In Brazil, the incidence was 95.1 per 100,000 inhabitants, while in Denmark the rate was 315.9 [5]. While individual rates per country are decreasing, the overall impact of hip fractures will increase due to the aging population, causing a greater morbidity burden in countries with lower socioeconomic resources and lower quality of life [4,6]. This is compounded by the fact that the increase in hip fracture prevalence may be associated with risk factors such as high BMI, lower physical activity, and increased smoking in the population [7].

The World Health Organization (WHO) anticipates an increase in hospital demand for hip fractures, estimating that cases could reach 6 million by 2050 [8]. It is one of the leading causes of hospitalization among older adults worldwide [2]. Although there is no evidence of a hospital stay target, some studies indicate that the average hospital stay ranges from approximately 9.8 to 22.4 days, depending on the postoperative treatment received and patient characteristics [9,10], and mortality is associated with a longer average hospital stay of 22.6 days [9]. Additionally, the preoperative period should be 24 to 48 h from hospital admission, as soon as it is safe for the patient [11], although a Mexican report indicates that only 7.6% of patients undergo surgery within the first 36 h [12].

Assessing the determinants of length of hospital stay (HS) in older adults with hip fractures is crucial to optimizing clinical care, improving health outcomes in this vulnerable group, and developing effective plans to reduce hospitalization time [13,14]. A prolonged HS in this situation has significant repercussions, including an increased risk of complications such as nosocomial infections, chronic functional impairment, and thromboembolic events [14,15]. At the same time, a prolonged preoperative period (>48 h) has been associated with a progressive increase in in-hospital mortality at 3 days [16], as well as infectious complications that prolong hospital stay [17,18]. In addition, prolonged hospitalization can increase costs for both the healthcare system and the patient [13]. These costs are comparable to those of other diseases with high hospitalization rates, such as cardiovascular diseases. However, it is likely that the social costs, such as the emergence of new comorbidities, sarcopenia, poor quality of life, disability, and increased mortality, are even higher [19]. This highlights the importance of addressing this problem in a timely and accurate manner to improve clinical outcomes and resource management efficiency.

In Peru, the incidence of hip fractures is high, affecting mainly older adults with an average age of 66 years, 71% of whom are women. Furthermore, research reveals that pathological antecedents like diabetes mellitus, cardiovascular diseases, neurological deficits, dementia, and poor nutritional status contribute to up to 77% of these fractures [2]. Peruvian Social Health Insurance estimates that hip fractures affect between 12% and 16% of women over 50 each year [20].

The resolution of hip fractures necessitates a multifactorial approach, including the administrative management of hospital services [21]. Most affected patients have a history of conditions such as diabetes mellitus, cardiovascular disease, neurological deficits, dementia, and poor nutritional status; this pathology extends beyond orthopedic problems [22,23,24]. These comorbidities impact areas such as internal medicine, rehabilitation, social work, and healthcare economics, underscoring the need for a holistic approach to patient management [25,26].

However, the evidence on the determinants of length of HS in older adult hip fracture patients remains insufficient, as previous studies had several limitations. First, many of these studies have small sample sizes [2]. Furthermore, the absence of key variables linked to HS duration, like surgery time and hospital care factors, results in information bias [27]. Also, in Peru, information on factors influencing the length of HS for hip fracture patients is scarce.

The aim of this research was to analyze the determinants of length of HS in older adult hip fracture patients in a hospital in the Lambayeque region of Peru during 2017–2019.

## 2. Materials and Methods

### 2.1. Study Design

An observational study based on a secondary data analysis of an existing database was conducted in a hospital in the Lambayeque region, Peru, during the years 2017–2019. The primary study aimed to evaluate preoperative complications in patients over 60 years of age with hip fractures, excluding those with multiple fractures, pathological fractures due to cancer or osteomalacia, and those whose data had been collected by the orthogeriatric unit. For the present study, we reanalyzed that database, with hospital length of stay as the outcome, and included an appropriate time frame and a sufficient sample size, monitored by the hospital’s orthogeriatric unit, which allowed for adequate data collection. The database can be found under open access.

### 2.2. Population, Sample, and Sampling

The study population consisted of adults over 60 years of age with a diagnosis of hip fracture treated at the Almanzor Aguinaga Asenjo Hospital, Peru, during the years 2017–2019, as registered in the original database. The sample size of the first study consisted of 432 patients. In the secondary analysis, the sample size was determined using the mean difference and standard deviation of the pre-surgical time variable, which was 4.2 ± 5.1 days for HS of 11–14 days and 2.9 ± 2.3 days for HS longer than 14 days, resulting in a sample size of 294 [28]. A sample size formula was used in Stata v.16 following specific variables such as means and standard deviations of two populations with and without the outcome, in addition to a 95% confidence level and 80% power [29]. Having a population in the first study that was larger than the sample size allows us to work properly with variables containing missing data; finally, a sample of 399 patients was drawn using non-probabilistic convenience sampling.

### 2.3. Eligibility Criteria

Medical records of patients aged 60 years or older with a definitive diagnosis of hip fracture, including periprosthetic fractures, were incorporated. This group was selected by means of convenience sampling that met the criteria for inclusion (clinical records corresponding to the period from June 2017 to January 2019) and exclusion (patients under 60 years of age, those with concurrent procedures, pathological hip fractures by clinical diagnosis, those who opted for voluntary discharge, patients who died before discharge, or those who did not undergo surgery).

### 2.4. Variables

The dependent variable was HS, operationally defined as days of stay from admission to hospitalization to discharge. A prolonged HS was also defined as a stay lasting ≥9 days; this categorization can be based on the fact that the presence of morbidity beyond the 8th day of hospitalization increases the length of hospital stay [30]. Although this is the description from the surgical service of the originating hospital, it is not defined as a clinical standard; therefore, the variable was taken in its categorical form for variable comparison and in its numerical form for analysis. We consider it important to note that, due to the lack of a standard definition for this variable, we analyzed it in detail as a numerical variable to meet the study’s objective. The independent variables were age, sex, and preoperative time. Functional Ambulation Category (FAC scale) is a category that evaluates the patient’s ambulatory capacity, with a score ranging from 0 to 5. “Mental Red Cross” (MRC scale) refers to an index that evaluates the patient’s cognitive status, and was validated by the Red Cross Hospital in Madrid [31]. It covers a score ranging from 0 to 5 degrees: Completely normal = 0. Slight disturbances such as disorientation in time, but can maintain a conversation correctly = 1. Disorientation in time, and conversation is possible but not perfect, knows people well, even though they sometimes forget things, personality disorders, occasional incontinence = 2. Disorientation, it is impossible to have a logical conversation, people are confused, clear mood disorders, frequent incontinence = 3. Disorientation, clear mental alterations, habitual or total incontinence = 4. Finally, advanced dementia with a vegetative state, with or without episodes of agitation, and total incontinence = 5. The Barthel index of basic activities of daily living is an index that evaluates the patient’s activities of daily living, with a score ranging from 0 to 100. Comorbidities are defined as the number and type of comorbidities present in the patient. These were grouped into diabetes, cardiovascular, cerebrovascular, osteoarticular, neurological, hepatic, renal, cancer, malnutrition, and others. Polypharmacy is defined as the consumption of five or more medications; geriatric syndromes refer to the number and the main geriatric syndrome present before hospital admission, like dementia, visual deprivation, falls, and depression. Laboratory analysis refers to measurements taken prior to surgery within the first 48 h of hospital admission, and the following were studied: hemoglobin, creatinine, urea, and basal glucose. Trauma diagnosis refers to the type of fracture defined by the traumatologist; the reason for surgical delay refers to the reason that led to the delay of the surgery, whether medical or administrative, such as medical complications, waiting for a hospital bed, lack of surgical materials, or delayed surgical scheduling. Preoperative complications refer to any medical complication that occurs before surgery and is evaluated from the time of admission to the emergency room. Additionally, variables such as time to emergency refer to the time from the fall to the patient’s arrival at the emergency unit. Emergency stay refers to the time from arrival at the emergency department to hospitalization.

### 2.5. Procedures and Techniques

In order to obtain the data, the records notebook and data collection form were requested from the Orthogeriatric Unit that carried out the follow-ups, from which the medical records of older adult patients with a diagnosis of hip fracture treated during the period 2017–2019 were selected. This unit was composed solely of geriatrics specialists who used the consultant model; the evaluation was conducted from the emergency department and did not include post-surgical hospital rehabilitation.

The data collection form of the Orthogeriatric Units was designed to collect information such as socio-demographic aspects (age, sex), clinical aspects such as the trauma diagnosis of the type of hip fracture, geriatric syndromes, comorbidities, FAC scale, Barthel Index (instrument that calculates the person’s capacity to carry out basic activities of life), MRC scale (physical and functional estimation subscale that indicates the index of cognitive status), polypharmacy (number of habitual drugs greater than or equal to 5 drugs), laboratory tests such as glucose, urea, creatinine, serum albumin, prothrombin time hemoglobin, white blood cell count, platelet count and urine culture, and the date of fracture, date of emergency admission, date of hospitalization, date of surgery and pre-surgical complications related to the patient during their HS.

Patients with an HS of ≥9 days were classified as having an extended HS; this time period was chosen based on a previous study conducted at the same hospital [32].

### 2.6. Data Analysis Plan

The statistical analysis and table preparation were carried out using Stata version 17. The clinical characteristics of the patients were described using frequency distribution tables, measures of central tendency (mean), dispersion (standard deviation), and interquartile range according to normality criteria. The Shapiro–Wilk normality test was performed to identify variables that did not follow a normal distribution, and a bivariate analysis with parametric and non-parametric tests was also conducted with a *p*-value < 0.05. Subsequently, a Poisson regression analysis was conducted using both a crude model and an adjusted model. Considering the assumptions of linearity, the variables that were associated in the simple model with a *p* < 0.05 were included in the adjusted model, previously meeting the assumptions of multicollinearity and equidispersion. Since the assumptions were not met, a negative binomial regression was used. Incidence rate ratios (IRR) and their 95% confidence intervals (CI) were used.

### 2.7. Ethical Considerations

The present study was approved by the Institutional Research Ethics Committee of the Faculty of Medicine of the Universidad de San Martín de Porres (No. 050-2022-CIEI-FMH-USMP), as well as by the Research Ethics Committee of the Hospital Almanzor Aguinaga Asenjo (No. 083-CIEI-RPLAMB-ESSALUD-2022). Since the study obtained its information from medical records, informed consent was not required.

## 3. Results

### 3.1. Epidemiological Characteristics and Hospitalization Times of the Population

**Figure 1:**
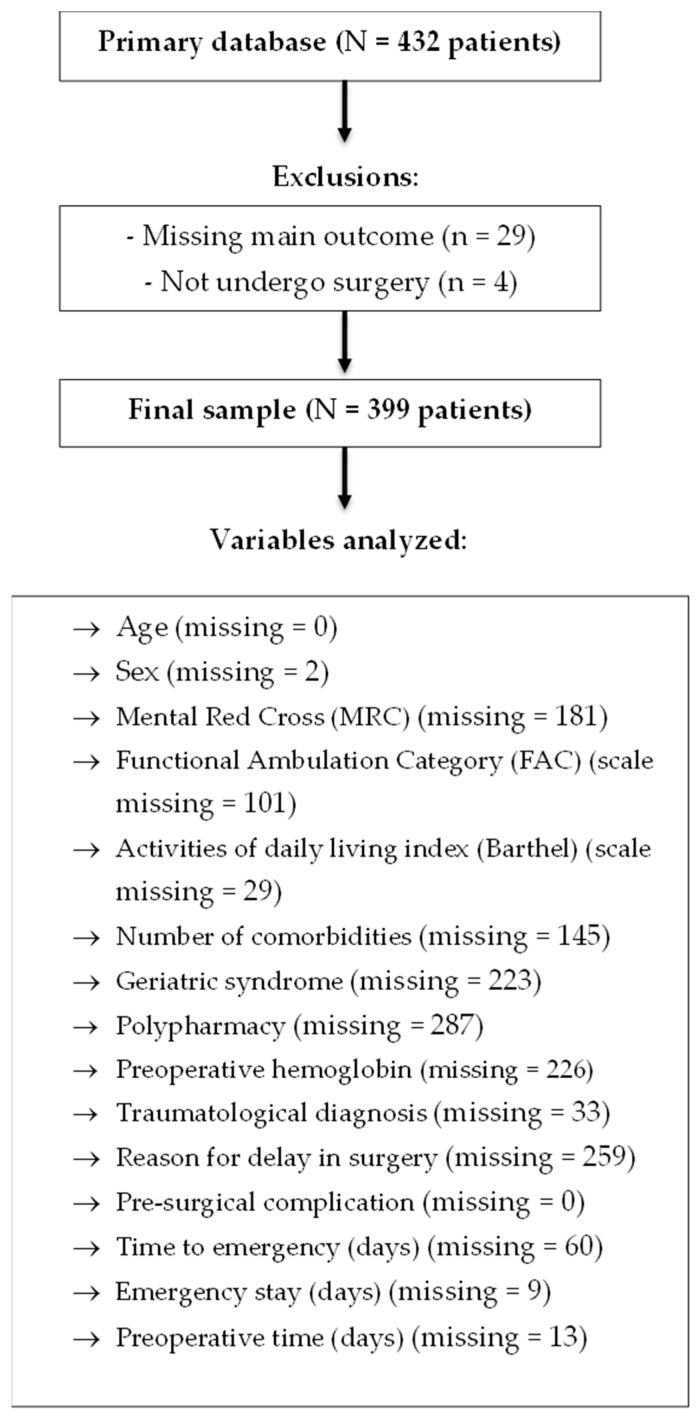
Selection flowchart and variables.

The analysis of 399 patients was performed after excluding 29 patients for not having the dependent variable of HS (of these patients, there were 14 deaths) (Figure 1). It was found that the minimum age was 60 years and the maximum age was 100 years, with most patients being women (63.7%). The patients had a median of three geriatric syndromes and two comorbidities at the time of hospital admission. In addition, 3% of patients had liver disease, 5% had renal disease, 4% had cancer, 4% had malnutrition, and 18% had other diseases. Additionally, 46 patients (21.2%) were identified as having an MRC scale score of 3 degrees or more. The average preoperative hemoglobin level was 11 g/dL, with a minimum value of 4.6 g/dL; the average glucose level was 131 mg/dL; the average creatinine level was 0.76 mg/dL, with a maximum value of 14.2 mg/dL; and the average urea level was 47.69 mg/dL, with a maximum value of 191 mg/dL. The most frequent pre-surgical complications were urinary tract infection, 40 (42.1%); pneumonia, 17 (17.9%); delirium, 12 (12.6%); sepsis, 9 (9.5%); and pressure ulcer, 9 (9.5%). In patients with prolonged HS, which was defined as ≥9 days, a higher frequency of MRC grade 3 or higher, considered severe dementia, was found, as well as a higher frequency of polypharmacy, a greater number of geriatric syndromes such as depression and visual impairment, and more pre-surgical complications (Table 1).

**Figure 2:**
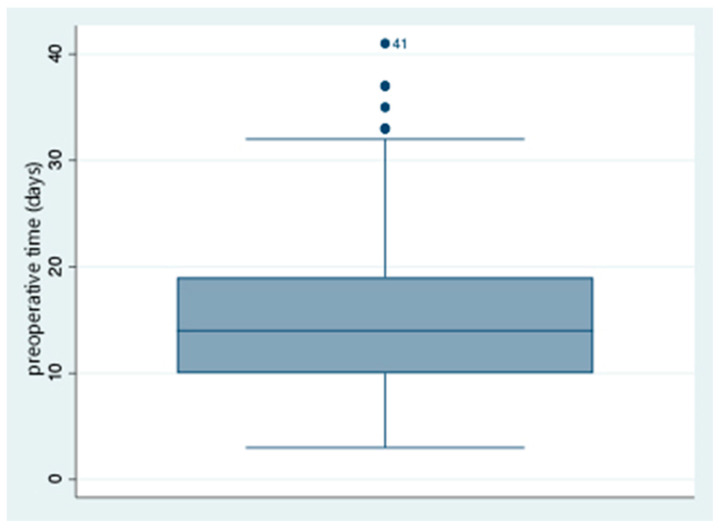
Box graph of preoperative time.

In relation to the hospital time indicators, the minimum preoperative time is 3 days, and the maximum is 41 days (Figure 2); the maximum emergency arrival time is 100 days, and the minimum was less than 1 day; and the minimum emergency stay was less than 1 day, and the maximum was 26 days. We examined the relationship with HS, finding that emergency stays and preoperative time were associated with prolonged stays (*p* < 0.001) (Table 2).

**Figure 3:**
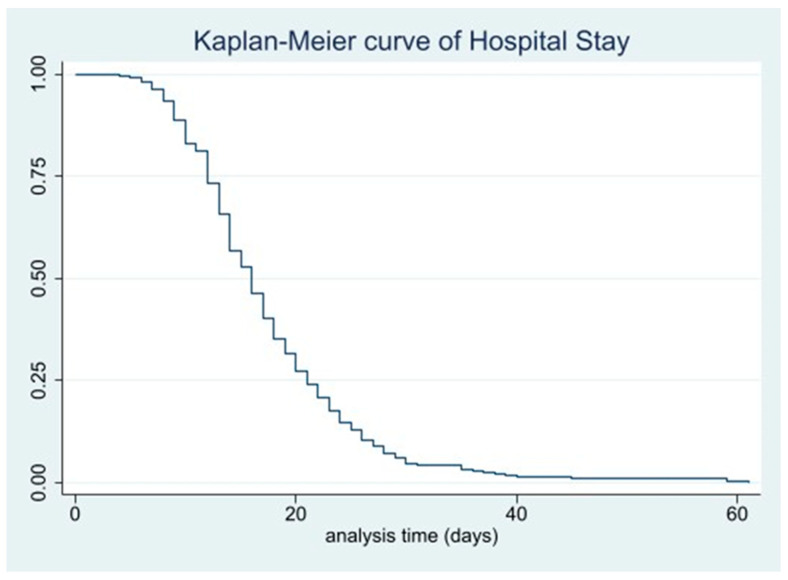
Kaplan–Meier curve of hospital stay.

**Figure 4:**
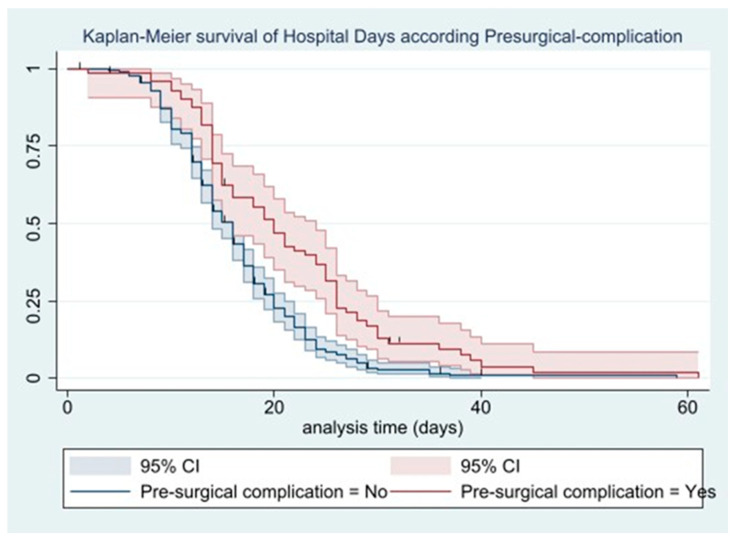
Kaplan–Meier survival of hospital days according to presurgical complication.

The Kaplan–Meier curves show that at 9 days of hospitalization, 88.6% of patients (95% CI = 85.1–91.4) remained hospitalized, with 370 patients at risk; at 15 days, 52.7% (95% CI = 47.6–57.5) remained hospitalized, with 220 patients at risk (Figure 3). Among patients with preoperative complications, 62.5% (95% CI = 50.3–72.5) remained hospitalized after 15 days, with 50 patients at risk, compared with 50.5% (95% CI = 44.9–55.9) of those without preoperative complications, with 170 patients at risk (Figure 4).

Variables including FAC scale (25.3%), number of comorbidities (36.3%), types of comorbidities (more than 50% missing data), geriatric syndromes (55.9%), the MRC scale (45%), and polypharmacy (72%) and all laboratory analyses had more than 50% missing data. Hospital variables such as time in the emergency room (15%), length of stay in the emergency room (2%), preoperative time (3.2%) and reason for delay in surgery (64.9%) also had missing data. For a more in-depth analysis of the raw data using crude and adjusted regression, the numerical and categorical variables identified as related in the bivariate analysis underwent simple imputation using means and medians depending on whether the variables followed a normal distribution. We considered it necessary not to use the polypharmacy variable or laboratory analyses such as glucose, urea, and creatinine due to the large amount of missing data that would hinder a proper analysis. We preferred to perform the analysis of hemoglobin levels, most frequent comorbidities, types of geriatric syndromes, and the MRC, as these are clinically important variables, even though simple imputation may alter the results.

### 3.2. Simple Poisson Regression Analysis

We used a Poisson regression analysis to evaluate the association between the described independent variables and HS; this variable was taken in its numerical form with the intention of assessing its relationship naturally with the other variables and avoiding categorical description due to the lack of a clear definition of prolonged hospital stay. An association was found in a first simple model between the clinical variables: age, female sex, number of comorbidities and multimorbidity, Mental Red Cross scale, Barthel index, FAC scale, number of geriatric syndromes, type of traumatic diagnosis, reasons for surgery delay, and preoperative complications with HS (*p* < 0.05). In turn, an association was also found between hospital stay variables—such as emergency department length of stay and preoperative time—and HS (*p* < 0.001) in a second model. Among the ambulation variables measured by FAC, an association was found with all categories and HS (*p* < 0.0001). In the cognitive impairment variable measured with the Mental Red Cross scale, an association was found between the categories of some memory problems, severe memory and orientation impairment, advanced dementia, and HS (*p* < 0.05). No linear relationship was found between age, Barthel, and emergency stay with HS, so they were not included in the adjusted model. No association was found between preoperative hemoglobin levels and hospital stay.

### 3.3. Adjusted Model Regression Analysis 

A Poisson regression analysis was conducted with an adjusted model for the variables that were significant (*p* < 0.05) in the crude model. It was decided to divide them into a clinical variables model, which included sex, multimorbidity, Mental Red Cross index, FAC scale, number of geriatric syndromes, reason for delay, and preoperative complication. In the second model of hospital times, emergency stay, and preoperative time were included. A third model was developed that included all variables. In the first model, multicollinearity was found between the number of geriatric syndromes and the FAC variables due to a variance inflation factor (VIF) > 6, so they were not included in the regression. A *p*-value < 0.05 was found for the equidispersion assumption, so Poisson regression could not be used, and negative binomial regression was chosen instead. The analysis found that, in the first adjusted model, advanced dementia as measured by the MRC scale (IRR = 1.82; 95% CI = 1.03 to 3.23) and preoperative complications (IRR = 1.56; 95% CI = 1.3 to 1.86) were associated with an increased rate of hospital length of stay, adjusted for sex, number of geriatric syndromes, multimorbidity, traumatic diagnosis, and reason for surgical delay (*p* < 0.001). In the second model, no multicollinearity was detected, and a Poisson analysis could be performed because equidispersion was plausible. In this second adjusted model, it was found that each additional day of preoperative time was associated with an increase in the rate of hospital stay days (IRR = 7.44, 95% CI = 6.96–7.96), adjusted for emergency admission. A third global model including all variables was fitted, in which multicollinearity was detected for the preoperative time variable (VIF > 6), so it was not included in the final regression. A *p*-value < 0.05 was found for the equidispersion assumption, so negative binomial regression was also chosen. Therefore, in the overall model, advanced dementia as measured by the MRC (IRR = 1.82, 95% CI = 1.02–3.23, *p* < 0.04) and the presence of preoperative complications (IRR = 1.56, 95% CI = 1.30–1.86, *p* < 0.04) were found to be associated with longer hospital stays, adjusted for clinical and hospital variables (Table 3).

## 4. Discussion

### 4.1. Average Hospital Stays of Older Adult Hip Fracture Patients

It was found that the average HS of older adult patients with hip fractures treated in a tertiary hospital in the region of Lambayeque, Peru, was 17 days. There are similar results in the study by Marufu T, which uses data from three hospitals in the United Kingdom, where the average HS was 16.9 days [30]. This is explained by the fact that HS in these hospitals mainly takes into account post-surgical stays. This differs from the findings of several studies, such as that of Nikkel et al., in which in the US population the average stay was 8.1 days [28], due to the fact that the destinations after discharge from the surgical unit were in-hospital rehabilitation, specialized nursing centers, and cancer centers, among others, as in the study by Fan T. in the Chinese population, in which the median HS was 4.9 days [27].

The median HS was similar to that in a previous study conducted in the surgical service of the same hospital. In that study, it was found that factors such as the scheduling of surgical procedures, having an “acquaintance” within the health system, the health conditions of the patients on admission, and the lack of specialists in the service are associated with a prolonged HS [32].

It is known that prolonged hospital stays can cause complications such as nosocomial infections, prolonged use of catheters [33], weakness and muscle atrophy induced by immobility [34], and delirium [35], which can increase the need for care. Moreover, it increases the risk of mortality by 32% at 30 days [28] and is considered a predictor of mortality at one year along with advanced age [9]. During the last decades, significant improvements have been seen in hospital stay standards, as in Canada, during the years 2004–2012, there were higher probabilities of discharge within the first 30 days, and higher probabilities of surgery within the first 24–48 h and between the 1st and 4th postoperative days [36]. Additionally, orthogeriatric services in countries such as Colombia report improvements in hospital stays from 17 to 10 days, and in Mexico [37], reductions of three days in hospital length of stay have been reported [38].

And this is due to the improvement of management policies for this condition in different countries, adherence to international clinical care standards, and orthogeriatric care, which has been determined to improve hospital stays [39]; this faces many limitations in its implementation in our Peruvian healthcare system.

### 4.2. Dementia and Length of Hospital Stay

Cognitive dysfunction represents a significant risk factor for hip fracture, also negatively influencing the recovery process due to the increased development of complications and patient confusion. In our study, advanced dementia as measured by the MRC scale is associated with a 1.8-fold increase in hospital length of stay in both the clinical variables model and the combined model. However, Rasu R. et al. found that patients with dementia and an unknown diagnosis of osteoporosis have a shorter HS [40], in contrast to multiple reports indicating a prolonged HS associated with dementia in older adults [41,42,43,44]. This is because advanced dementia is associated with a greater impairment of physical functions, increased dependence, a higher risk of delirium, and other geriatric syndromes that can increase preoperative complications and hospital length of stay, although there may be other confounding variables such as frailty—which have not been measured and have been shown to increase hospital length of stay [45]—in addition to a specific correlation with cognitive impairment in the hospital setting [46].

### 4.3. Preoperative Complications and Length of Hospital Stay

Our study found that preoperative complications are associated with a more than 1.5-fold increase in hospital length of stay in both the adjusted clinical variables model and the combined model. This is related to other studies where preoperative complications such as delirium have been studied, which have been associated with a hospital stay longer than 20 days, in addition to mortality and hospital readmissions [47]. Moreover, in the South African population, the presence of pressure sores in patients with hip fractures increased their hospital stay [48]. Pre- or postoperative complications in this population have been decreasing over the past 20 years, according to a Chinese study, with a more notable reduction in pneumonia, cardiovascular events, and respiratory failure [49]. This may be due to a multidisciplinary approach for this population, as geriatric teams are recognized to be more effective in addressing morbidities [50].

### 4.4. Hospital Times and Length of Hospital Stay

Our study found in its preliminary model that each additional day of preoperative time is associated with a more than sevenfold increase in hospital length of stay, after adjusting for emergency department stay. This association could not be replicated in the final model of clinical and hospital variables, which may be explained by the potential effect of confounding and intervening variables—such as preoperative complications or a history of dementia—on the time to surgery and, consequently, on length of stay, as well as by a possible spurious mathematical relationship between the two variables. However, the association between preoperative time and length of hospital stay is plausible and could be explained by several reasons. First, a prolonged preoperative period could also increase the likelihood of preoperative complications. During this period, patients may experience a deterioration in their general condition due to prolonged immobilization, chronic pain, or increased uncontrolled comorbidities, which could complicate surgery and the postoperative period [51]. Second, prolonged preoperative time may affect the planning and timing of surgery. Delays in surgical scheduling can lead to a greater backlog of pending interventions, which can result in an overload of medical and surgical staff and, thus, less efficient care. This can prolong the total hospitalization time while complications are resolved or while waiting for the right time to perform surgery, which is why it is considered a critical process indicator of therapeutic success in hip fracture management [52]. Third, an extended preoperative time may mean increased exposure to hospital risk factors, such as nosocomial infections like urinary tract infections or pressure injuries [17,18]. A prolonged stay before surgery may increase the possibility of adverse events related to hospitalization, which in turn may extend HS due to the need to manage these complications.

Although no studies have been found in which the main finding for prolonged HS is preoperative time, Kristan A et al., in a multiple regression analysis, found that patients operated on after 48 h (delayed surgery) had an additional 7.3 h of HS for every 10 h of delay in surgery. In addition, factors such as the American Society of Anesthesiologists (ASA) score, anticoagulation therapy, and type of operation did not increase HS [41]. Likewise, Schweller et al. found that increased preoperative time has a significant association (*p* < 0.001) with increased HS in patients [14]. In addition, Varady et al. found in their study that delay in surgery was associated with an increased risk of prolonged postoperative length of stay (*p* < 0.001) [53].

The optimization of preoperative time involves not only timely, comprehensive evaluation of the patient for scheduling but also early arrival at emergency services. This has been shown to reduce HS and optimize the use of economic resources. Chen X et al., in their research, analyzed the waiting times prior to admission to emergency services, classifying them into less than 8 h, 8 to 24 h, and more than 24 h. Patients with waiting times longer than 24 h showed a prolonged HS and higher costs during their HS [33]. However, the time prior to admission was not associated with hospital stay in our study.

This describes the lack of an efficient local comprehensive hip fracture management program that could reduce hospital stays and, above all, ensure adequate management of older adults with a hip fracture. Several studies have shown that comprehensive management, or the intervention of an orthogeriatric service exclusively for hip fracture patients, reduces 1-year mortality, length of HS, and additional rehabilitation requirements [54]. Therefore, a comprehensive program with a multidisciplinary approach significantly improves outcomes [55,56].

### 4.5. Other Determinants of Hospital Stay

According to the results of this study, in the simple model regression analysis, the number of comorbidities and geriatric syndromes and multimorbidity were associated with hospital stay. However, in our adjusted analysis, the multimorbidity variable did not show a statistically significant relationship. Several studies point to a significant relationship between patients with comorbidities and hip fractures, which is associated with increased HS. Lim, J. states that the length of HS for hip fracture patients depends on the outcome of treatment, the number of comorbidities, and the availability of hospital beds. Comorbidities associated with prolonged HS include hypertension (mean 25.54 days), peptic ulcers (45.83 days), coagulopathy (40.89 days), and alcohol abuse (40.75 days), all with a significance level of *p* < 0.05 [57]. The high percentage of missing data on comorbidities is likely to influence the accurate measurement of the strength of association. However, these findings suggest the need to adopt diverse healthcare approaches depending on each patient’s comorbidities or an integrated approach to improve medical care, optimize preoperative time, and reduce prolonged HS [58].

When we evaluated mobility variables such as the FAC scale and basic activities such as the Barthel index in the crude model, we observed that all categories of mobility limitation and alteration of basic activities were associated with hospital stay; however, no association was found in the adjusted model. In the Schneider AM study, it was observed that patients with independent functional ability had a prolonged HS of 70.1% (*p* < 0.0001) [59]. Although our study found no association in the adjusted model between prior ambulation and dependence with hospital stay due to a high percentage of missing data, it is likely that prior functional dependence leads to a higher risk of pre- and postoperative complications and a longer hospital stay; additionally, these dependent patients may be discharged earlier to their geriatric residences due to limited rehabilitation options in hospitals that provide post-acute care continuity, therefore reducing their hospital stay.

### 4.6. Practical Implications of Findings in Geriatrics

The findings of this study have important practical implications for the care of geriatric hip fracture patients. First, the identification of preoperative time as a determinant of the length of HS highlights the need to optimize preoperative processes [17]. Implementing protocols that reduce the waiting time for surgery through better coordination between emergency and operating room services could significantly shorten HS, improve patient recovery, and reduce hospital costs [60,61].

Additionally, the results highlight the importance of a multidisciplinary approach in the management of geriatric patients. Comprehensive care that addresses not only hip fractures but also comorbidities, geriatric syndromes such as dementia, and the prevention of preoperative complications is relevant to improving clinical outcomes. Comorbidity management and early rehabilitation programs have been shown to help reduce complications and accelerate recovery, resulting in shorter HS and improved quality of life for patients [62]; therefore, their application in Latin American countries is important.

Furthermore, these findings suggest the need for policies and strategies that promote appropriate management through procedural indicators in geriatric hip fracture patients. Therefore, optimizing pre-operative times and adopting a comprehensive, multidisciplinary approach to geriatric care will not only improve hospital efficiency and reduce costs but also ensure better care and health outcomes for older adults [61].

### 4.7. Limitations and Strengths

The study had some limitations. First, a considerable proportion of data was missing for key clinical variables, such as ambulation measured using the FAC scale, multimorbidity, and geriatric syndromes, as well as hospital variables such as types of delay (e.g., lack of surgical scheduling and lack of surgical supplies), which may have lost their strength of association in the adjusted models due to the missing data. Although the imputation methods were applied, these gaps may have influenced the absence of statistically significant associations in the crude and adjusted model, in addition to bias or errors of interpretation, despite such associations having been demonstrated in other studies [63]. Secondly, the secondary data analysis design, based on an existing database, limits the availability of certain variables and may be subject to selection bias, which could affect the validity of the findings. Thirdly, the non-random sampling used in the study could influence the results by increasing the risk of selection bias and limiting the sample’s representativeness. Therefore, although the findings are valid for the group studied, they should be interpreted with caution in other populations or contexts. Fourth, although adjustments were made in the regression models to control for potential confounding factors, confounding factors not adequately measured due to missing data, such as hemoglobin levels [64], urea [27], polypharmacy, and use of anticholinergic medications that could have influenced the results cannot be completely ruled out [65]. Fifth, although the exclusion of deceased patients accounts for 3% of the total study population and is in line with international mortality rates, and the strength of association of the resulting variables is not minimal, there could be bias by underestimating or overestimating the results by not considering deceased patients. Finally, the period during which the 2017–2019 data were recorded could affect some hospital variables, since the hospital system may have been better organized, allowing for shorter times and fewer surgical delays.

Despite these limitations, the study presents important strengths that reinforce the relevance of its findings. First, the detailed analysis of the variables related to the length of hospital stay, including preoperative time and geriatric clinical conditions, offers a comprehensive view of factors influencing hospitalization in older patients with hip fractures Secondly, the use of regression models allows for a rigorous evaluation of the associations between independent variables and the length of hospital stay, providing precise estimates of the effects. Thirdly, the study focuses on a specific population in a tertiary hospital in northern Peru, providing a relevant local perspective for the context of geriatric care in this context, helping to identify region-specific factors and contributing to the improvement of hospital management for older adults.

## 5. Conclusions

The average hospital stay for older patients with hip fractures treated at a tertiary hospital in the Lambayeque region of Peru was 17 days. Most of these patients were women (63.7%) aged 60 to 100 years. The main factors associated with the duration of hospital stay were advanced dementia as measured by the MRC and the presence of preoperative complications.

## Figures and Tables

**Table 1:** Clinical characteristics of older adult patients with hip fractures according to prolonged hospital stay in a hospital in the Lambayeque region of Peru during 2017–2019.

Variable	Total N = 399	Prolonged Hospital Stay	
		Yes	No
**Age**	82.25 ± 8.18	81.99 ± 8.23	85.54 ± 6.86
**Sex** ⁂			
Male	144 (36.3)	131 (35.6)	13 (44.8)
Female	253 (63.7)	237 (64.4)	16 (55.2)
**Mental Red Cross (MRC)** ⁂			
Normal	70/218 (32.1)	66 (32.4)	4 (28.6)
Some memory disorder	52/218 (23.9)	49 (24)	3 (21.4)
Memory and orientation impairment	50/218 (22.9)	47 (23)	3 (21.4)
Severe memory and orientation impairment	37/218 (17)	34 (16.7)	3 (21.4)
Dementia and incontinence	8/218 (3.7)	7 (3.4)	1 (7.1)
Advanced dementia	1/218 (0.5)	1 (0.5)	0 (0)
**Functional Ambulation Category (FAC) scale** ⁂			
Walks with extensive assistance from 1 person	34/298 (11.4)	29 (10.5)	5 (21.7)
Walks with light physical contact	47/298 (15.7)	44 (15.9)	3 (13)
Walks with supervision	55/298 (18.4)	50 (18.1)	5 (21.7)
Independent ambulation on level ground	52/298 (17.4)	48 (17.4)	4 (17.4)
Walks independently on level ground and up stairs	51/298 (17.1)	49 (17.8)	2 (8.7)
Normal ambulation	59/298 (20.1)	53 (20.1)	6 (17.1)
**Activities of daily living index (Barthel)** ⁂	80.36 ± 23.22	80.96 ± 22.62	72.50 ± 29.47
**Polypharmacy** ⁂			
No	9/112 (8)	8 (7.5)	1 (16.7)
Yes	103/112 (92)	98 (92.5)	5 (83.3)
**Geriatric syndrome** ⁂	3.36 ± 2.47	3.41 ± 2.5	2.67 ± 1.92
Dementia or cognitive impairment	32/61 (52.5)	29 (50.9)	3 (75)
Visual deprivation	9/61 (14.8)	9 (15.8)	0 (0)
Falls	8/61 (13.1)	8 (14)	0 (0)
Depression	12/61 (19.7)	11 (19.3)	1 (25)
**Comorbidities**	1.81 ± 1.26	1.8 ± 1.26	1.92 ± 1.26
Diabetes	15/163 (9.32)	14 (9.03)	1 (14.29)
Cardiovascular disease	59/163 (36.65)	57 (36.77)	2 (28.57)
Cerebrovascular disease	5/163 (3.11)	5 (3.87)	0
Osteoarticular disease	15/163 (9.32)	14 (9.03)	1 (14.29)
Neurological disease	12/163 (7.45)	12 (7.74)	0
**Laboratory analysis**			
Preoperative hemoglobin	10.99 ± 1.91	11.1 ± 1.88	10.2 ± 2.03
**Traumatological diagnosis**			
Neck fracture	108/366 (29.5)	103 (30.2)	5 (20)
ITT Fracture I–II	97/366 (26.8)	86 (25.2)	11 (44)
ITT Fracture III–IV	120/366 (32.5)	114 (33.4)	6 (24)
Subtrochanteric fracture	41/366 (11.2)	38 (11.1)	3 (12)
**Reason for delay in surgery**			
Scheduling	76140 (54.3)	71 (54.2)	5 (55.6)
Waiting for a hospitalization bed	45/140 (32.1)	42 (32.1)	3 (33.3)
Surgical material	8/140 (5.7)	8 (6.1)	0
Medical complication	11/140 (7.9)	10 (7.6)	1 (11.1)
**Pre-surgical complication**			
No	326 (77.08)	301 (81.4)	25 (86.2)
Yes	73 (22.9)	69 (18.6)	4 (13.8)

Prolonged stay: ≥9 days. ITT: intertrochanteric fracture. ⁂ There are variables such as sex (2 patients) and geriatric assessment variables (number of comorbidities with 145 patients, Barthel index with 29 patients, MRC with 181 patients, FAC with 101 patients, geriatric syndrome with 223, and polypharmacy with 287) that have missing data.

**Table 2:** Indicators of hospital times in patients with hip fracture and prolonged hospital stay.

Variable	Total N = 399	Prolonged Hospital Stay		*p*-Value **
		Yes	No	
Time to emergency (days)	1 * (1-0)	1 * (2-0)	0.5 * (1-0)	0.408
Emergency stay (days)	3 * (5-2)	3 * (5-2)	3 * (5-1)	<0.001
Pre-operative time (days)	14 * (19-10)	14 * (19-11)	6 * (7-5)	<0.001

* Median; (): interquartile range P75–P25. ** *p* values were calculated using Mann–Whitney U analysis. Some variables had missing values (time to emergency with 60 patients, emergency stay with 13 patients, and pre-operative time with 13 patients).

**Table 3:** Clinical factors associated with hospital stay in older adult patients with hip fractures (n = 399).

Characteristics	Hospital Stay (HS)							
	IRR Crude	*p*-Value	IRR Adjusted (Model 1)	*p*-Value *	IRR Adjusted (Model 2)	*p*-Value *	IRR Adjusted (Model 3)	*p*-Value *
**Age**	**0.99**	**0.024**	**-**				**-**	
**Sex** ⁂								
Male	Ref.							
Female	**1.06**	**0.009**	1.04	0.508			1.04	0.508
**Comorbidities**								
Number of comorbidities	1.07	<0.001	**-**					
Cardiovascular disease	0.92	0.457	-					
Diabetes mellitus	0.92	0.303	-					
Multimorbidity **	**1.21**	**<0.001**	1.14	0.110			1.14	0.110
**Mental Red Cross (MRC)** scale ⁂		**0.021**						
Normal	Ref						Ref	
Some memory disorders	1.17	<0.001	1.16	0.058			1.16	0.058
Memory and orientation impairment	1.08	0.068	0.95	0.604			0.95	0.604
Severe memory and orientation impairment	1.12	0.019	1.06	0.504			1.06	0.504
Dementia and incontinence	1.09	0.336	0.76	0.128			0.76	0.128
Advanced dementia	2.46	<0.001	**1.82 (1.03–3.23)**	**0.04**			**1.82 (1.02–3.23)**	**0.04**
**Activities of Daily Living Index (Barthel)** ⁂	**0.99**	**0.005**	-	-			-	-
**Functional Ambulation Category (FAC) scale** ⁂		**<0.001**	**-**	-			-	-
Walks with extensive assistance from 1 person	1.28	<0.001						
Walks with light physical contact	1.26	<0.001						
Walks with supervision	1.35	<0.001						
Independent ambulation on level ground	1.28	<0.001						
Walks independently on level ground and up stairs	1.19	0.001						
**Geriatric syndrome** ⁂								
Number of geriatric syndromes	**1.05**	**<0.001**	**-**	**-**			**-**	**-**
Dementia			**-**				**-**	**-**
Visual deprivation	1.09	0.396	**-**				**-**	**-**
Falls	1.19	0.079	**-**				**-**	**-**
Depression	1.09	0.314	**-**				**-**	**-**
**Laboratory analysis**								
**Preoperative hemoglobin**	0.99	0.837						
**Trauma diagnosis**		**0.006**						
Cervical fracture	Ref						Ref	
ITT fracture I and II	0.99	0.858	1.07	0.333			1.08	0.333
ITT fracture III and IV	1.07	0.035	1.09	0.275			1.08	0.275
Subtrochanteric fracture	1.12	0.006	1.2	0.120			1.2	0.120
**Reason for delay in surgery**		**<0.001**						
Medical complication	Ref						Ref	
Waiting for a hospital bed	0.87	0.004	0.94	0.384			0.94	0.333
Lack of surgical material	1.19	0.036	1.06	0.719			1.05	0.275
Delayed surgical scheduling	1.21	0.006	0.81	0.108			0.81	0.120
**Pre-surgical complication**	**1.30**	**<0.001**	**1.56 (1.30–1.86)**	**<0.001**			**1.56 (1.30–1.86)**	**<0.001**
**Time to emergency (days)**	1.00	0.14			**-**	**-**	**-**	**-**
**Emergency stay (days)**	**1.03**	**<0.001**			1.01	0.257	**-**	**-**
**Pre-operative time (days)**	**1.05**	**<0.001**			**7.44 (6.96–7.96)**	**<0.001**	**-**	**-**

* *p*-value according to negative binomial regression. ** Presence of two or more comorbidities in the same person. ⁂ There are variables such as sex and geriatric assessment variables (Barthel index, MRC, FAC, geriatric syndrome, and polypharmacy) that have missing data. IRR crude: Incidence rate ratio. ITT: Intertrochanteric fracture.

## Data Availability

The datasets generated and/or analyzed during the present study are publicly available in the Figshare repository at the following link: https://doi.org/10.6084/m9.figshare.30226936.v1 (accessed on 28 September 2025).
